# Inducing Synergistic DNA Damage by TRIP13 and PARP1 Inhibitors Provides a Potential Treatment for Hepatocellular Carcinoma

**DOI:** 10.7150/jca.66020

**Published:** 2022-04-11

**Authors:** Haojun Xu, Zhijie Ma, Xiao Mo, Xiaoli Chen, Fanggui Xu, Fubing Wu, Hongjin Chen, Guoren Zhou, Hongping Xia, Chengfei Zhang

**Affiliations:** 1 School of Basic Medical Sciences &Key Laboratory of Antibody Technique of National Health Commission & Jiangsu Antibody Drug Engineering Research Center, Nanjing Medical University, Nanjing 211166, China.; 2 Sir Run Run Hospital, Nanjing Medical University, Nanjing 211166, China.; 3 Jiangsu Cancer Hospital & The Affiliated Cancer Hospital of Nanjing Medical University& Jiangsu Institute of Cancer Research, Nanjing 2100092, China.; 4 Department of Pathology, The first people's hospital of Foshan, Foshan 528041, China

**Keywords:** DCZ0415, TRIP13, H2AX, hepatocellular carcinoma, PARP1

## Abstract

Thyroid hormone receptor interactor 13 (TRIP13), an AAA-ATPase, participates in the development of many cancers. This study explores the function of TRIP13 and synergistic effects of TRIP13 and PARP1 inhibitors in hepatocellular carcinoma (HCC). The dose-dependent effects of TRIP13 and PARP1 inhibitors on HCC cells proliferation or migration were investigated by the CCK-8 and Transwell assays. Using siRNA or lentivirus to knock down TRIP13, we tested HCC cell and tumor growth *in vitro* and *in vivo*. The DNA damage caused by TRIP13 and PARP1 inhibitors was measured by the phosphorylation of H2AX, one of the DNA damage biomarkers. The phosphorylation of H2AX was increased after treatment with DCZ0415 or TRIP13 knockdown. Combining DCZ0415 with PARP1 inhibitor, Olaparib induced synergistic anti-HCC activity. We also found that the overexpression of TRIP13 is significantly associated with early recurrent HCC and poor survival. Up-regulation of TRIP13 in HCC was regulated by transcription factor SP1. In conclusion, our study demonstrated that DCZ0415 targeting TRIP13 impaired non-homologous end-joining repair to inhibit HCC progression and had a synergistic effect with PARP1 inhibitor Olaparib in HCC, suggesting a potential treatment of HCC.

## Introduction

Liver cancer is the third leading cause of cancer-related death globally, and its incidence is seriously increasing every year 1-3. Hepatocellular carcinoma (HCC), the most common primary malignancy in the liver, accounts for more than 90% of liver cancers 4. Current treatment options for HCC patients include surgical resection, orthotopic liver transplantation, radiofrequency thermal ablation, and targeted therapy 5-7. However, due to the aggressiveness and high postoperative recurrence rate of HCC patients, the prognosis is worse. The 5-year survival rate for HCC patients is less than 20% 8, 9. Therefore, it is urgent to identify new therapeutic targets and inhibitors, and explore the molecular mechanism of HCC progression.

Recently studies have demonstrated that the AAA-ATPase family is involved in various biological processes, primarily in DNA replication and protein folding 10. Thyroid hormone receptor interactor 13 (TRIP13), a member of the highly conserved AAA-ATPase family, has been shown an oncogenic gene 11, 12. TRIP13 regulates cell division during mitosis by remodeling the spindle assembly checkpoint (SAC) effector protein MAD2 into an inactive form 11. Moreover, TRIP13 mediates double-strand break repair through selective error-prone non-homologous end connections 13. It has been reported that TRIP13 mutation leads to the premature separation of chromosomal instability (CIN) and chromosome dysfunction, resulting in DNA aneuploidy 14. In addition, TRIP13 participated in the repair of chemotherapy resistance and cell transformation, further promoting the progression of head and neck cancer 15. Overexpression of TRIP13 is associated with poor prognosis in breast cancer 16, colorectal cancer 17 and glioblastoma 12. In addition, Zhu et al. reported that compared with normal liver tissue, the expression of TRIP13 was increased in HCC patients. Knockdown of TRIP13 inhibited the proliferation and migration process of HCC cells and attenuated HCC cell growth *in vivo* assay 18. Nowadays, Wang et al. reported that DCZ0415 was the small-molecule inhibitor targeting TRIP13, which could suppress multiple myeloma progression 19. However, the role of the DCZ0415 function and TRIP13 underlying mechanism in the development of HCC is still unclear.

Nowadays, drug combination is one of the important treatment options for HCC. PARP1, poly ADP-ribose polymerase I, was investigated to be highly expressed in HCC and became a promising target for cancer therapy 20-22. Olaparib is one of the PARP1 inhibitors. Increasing studies showed that Olaparib inhibits tumor growth of hepatoblastoma in patient-derived xenograft models and overcomes Sorafenib resistance through reshaping the pluripotent transcriptome in hepatocellular carcinoma 20, 23. In addition, a combination of Olaparib and NU7441 (a DNA-PKcs inhibitor) synergistically inhibits HCC growth by inhibiting PARP1, and cancels HR repair 24. TRIP13 knockout cells, one of the proteins involved in DNA damage repair, showed increased sensitivity to Olaparib 25. These researches indicated that Olaparib could be a potential treatment drug in the future.

In this study, we investigated the effects of DCZ0415 and TRIP13 on the proliferation, migration, and invasion ability of HCC cells and initially tested the mechanism of TRIP13. We tested inhibition levels of DCZ0415 with dose-dependence about HCC cells proliferation and invasion and found that TRIP13 was regulated by SP1 to promote non-homologous end joining (NHEJ) in HCC. Furthermore, we investigated that DCZ0415 increased the drug sensitivity of Olaparib to HCC cells.

## Materials and methods

### Cell culture and reagents

HuH7, HCCLM3 and Hep3B cell lines were obtained from American Type Culture Collection (ATCC) and were maintained in DMEM medium containing 10% fetal bovine serum (Sigma) and 1% Penicillin-Streptomycin (E607011, Sangon Biotech (Shanghai) Co., Ltd). DCZ0415, Mithramycin A, Olaparib were purchased from MedChemExpress (MCE). GAPDH and TRIP13 antibodies were obtained from Proteintech. SP1 and γH2AX antibodies were purchased from ABclonal. Goat Anti-Rabbit IgG (H+L) HRP and Goat Anti-Mouse IgG (H+L) HRP secondary antibodies were obtained from Bioworld.

### CCK8 assay

HuH7, HCCLM3 and Hep3B cells were seeded into 96-well plates at a density of 2,000 cells per well with different concentrations of DCZ0415 or/and Olaparib for 3 days. 10 μL CCK8 reagent (40203ES76, Yeasen) was added to each well, the absorption was detected at 450 nm after 1.5 h incubation.

### Colony formation assay

2,000 cells were seeded per well into a 6-well plate and treated with different concentrations of DCZ0415 for 15 days. After being fixed with 4% paraformaldehyde for half an hour, it was stained with 0.5% crystal violet for 30 min. Cells were photographed and counted by ImageJ software.

### Transfection assay

For siRNA transfection: small interfering RNA (siRNA) targeting different sequences were siTRIP13-1, forward sense 5'-GCUGGUAACCAAGAUGUUU-3', reverse sense 5'-AAACAUCUUGGUUACCAGC-3'; siTRIP13-2, forward sense 5'-CCCAUCGAUUUGAGUGCAU-3', reverse sense 5'-AUGCACUCAAAUCGAUGGG-3'; siSP1, forward sense 5′-GGUAGCUCUAAGUUUUGAU-3′, reverse sense 5′-AUCAAAACUUAGAGCUACC-3′. siRNA were synthesized by Genepharma (Shanghai, China) and transfected into HCC cells using ExFect^®^2000Transfection Reagent (purchased from Vazyme, Nanjing China) according to the manufacturer's protocol. After 72 h incubation, total RNAs and total proteins were extracted for further assays.

For overexpression plasmid, transfections: stably overexpressed TRIP13 cell lines and stably overexpressed SP1 cell lines were constructed by using pLenti-CMV-GFP-Puro empty-vector (purchased from Biogot Technology) embarked the sequences of TRIP13 or SP1. The stable cells were obtained by selecting with puromycin (2 μg/mL) for 7 days.

### Dual-Luciferase Reporter Assay

The mutant TRIP13 promoter was constructed by point mutation—the binding sites' sequence was CCGTTC instead of CCGCCC. To detect the effect of SP1 on exogenous TRIP13 promoter, a dual-luciferase reporter assay was performed. Cells were plated into a 12-well plate. The pGL3 vector, which contained both the firefly luciferase gene and the wild-type or mutant TRIP13 promoter, was co-transfected with the plasmid containing or not SP1 and the plasmid containing renilla luciferase. Cells transfected were harvest to be lysed after 36 h incubation. The relative TRIP13-luciferase activity was detected using the Dual-Luciferase® Reporter Assay System (Promega, USA), referring to the manufacturer's instructions.

### Quantitative real-time PCR

Total RNAs from HuH7, HCCLM3 and Hep3B cell lines were extracted using TRIzol reagent (Tiangen Biotech CO., LTD, Beijing, China). The cDNAs were synthesized by 5×All-In-One RT MasterMix Kit (ABMgood). All of the above assays were performed following the corresponding manufacturer's instructions. Quantitative real-time PCR was performed using a Hieff® qPCR SYBR Green Master Mix (Yeasen, Shanghai, China) on the Bio-Rad CFX96Real-time PCR instrument (Bio-Rad, USA). The primer sequences are as follows: TRIP13, forward 5′-CTGGAGGAAGAGACAGAAAACATAA-3′ and reverse 5′-GTTGTCATCACATAATCGAGGAGAT-3′; GAPDH, forward 5′-ACCCAGAAGACTGTGGATGG-3′ and reverse 5′-TTCAGCTCAGGGATGACCTT-3′. Relative expression was calculated using the 2^-ΔΔCt^ method.

### Western Blot assay

Total proteins from HCC cells were extracted using ice-cold RIPA buffer (50 mM Tris, pH 7.4, 150 mM NaCl, 2 mM EDTA, 0.5% Nonidet P-40) containing fresh protease and phosphatase inhibitors, and the protein concentration was measured using the BCA Protein Assay Kit (Beyotime). Proteins were denatured and separated by 10% SDS-PAGE gel, transferred onto nitrocellulose membranes, and blocked with 5% BSA. Followed by incubation with primary antibodies and corresponding secondary antibodies conjugated with HRP, protein bands were visualized with an ECL kit (Beijing Sage Creation). GAPDH was used as an internal control gene.

### Cell cycle and apoptosis assays

Cells were incubated with different concentrations of DCZ0415 for 24 h.

For cell cycle assay: cells were harvested, followed by washing twice with PBS, and were fixed overnight with 70% ethanol. Cell samples were stained by propidium iodide (PI), and the intensities were detected using flow cytometry (FACSVerse, BD, USA) by Analysis and Testing Center, Nanjing Medical University.

For apoptosis assay: supernatant DMEM medium and cells were collected, centrifuged 3 min, 800 g, and the pellets were washed twice by PBS. Cells were resuspended in 400 μL of precooled 1×binding buffer and stained by Annexin V-FITC/PI (Yeasen, Shanghai, China) for 30 min. Then cell suspension was detected on flow cytometry (FACSVerse, BD, USA) analysis and Testing Center, Nanjing Medical University.

### Transwell assay

For cell invasion: 30 μL diluted Matrigel (Corning, USA) was distributed into the membrane of the transwell insert, incubated at 37 ℃ for solidification. DMEM medium containing 20% FBS was added to the lower chamber. 12 h serum-starved cells were harvested and resuspended in serum-free DMEM medium; it was distributed 5×10^4^/ per 250 μL/per upper chambers. After 36 h incubation, the culture medium was removed, cells grown on the membrane of the transwell insert were washed twice by PBS and fixed in 4% paraformaldehyde for 30 min. After using cotton swabs to remove the cells grown in the upper chamber, the ones who grown on the lower chamber were stained with 0.1% crystal violet cells for 30 min, washed, and photographed under a microscope (Ts2, Nikon, Japan). Cells were counted by Image J software. For cell migration: the procedure was similar to the above cell invasion, just without the step for the matrigel.

### TUNEL (terminal deoxynucleotidyl transferase dUTP nick end labeling) assay

Cells were fixed with 4% paraformaldehyde and then treated with 100 μL proteinase K (20 μg/mL) for 5min. The cells were then stained with Alexa Fluor 488-12-dUTP Labeling Mix Recombinant TdT enzyme according to the manufacturer's instructions of TUNEL Apoptosis Detection Kit (Alexa Fluor 488) (Yeasen, Shanghai, China) and DAPI. Cells were then captured with the help of a fluorescence microscope (Ts2, Nikon, Japan).

### Immunofluorescence analysis

Cells were transfected with different siRNAs, incubated onto a confocal dish for 72 h. After washing twice by PBS, the cells were fixed with 4% paraformaldehyde for 30 min and permeabilized with 0.1% Triton-X for 5 min. Cells were blocked in 5% BSA for 1 h and then incubated with primary antibodies and corresponding secondary antibodies conjugated with Cy3 or FITC. After 20 min incubation with 4′,6-diamidino-2-phenylindole (DAPI, Sigma, USA), the samples were photographed under a fluorescence microscopy (Ts2, Nikon, Japan).

### Statistical analysis

In this study, each experiment was performed more than two or three times independently. Data were analyzed and expressed as mean ± SEM using GraphPad Prism 8 software. The Student's t-test (two-tailed) was used for comparison between groups. A one-way ANOVA test was used when three or more groups were compared. P-value <0.05 was considered statistically significant.

## Results

### TRIP13 inhibitor DCZ0415 suppressed HCC cell proliferation

**Figure 1:**
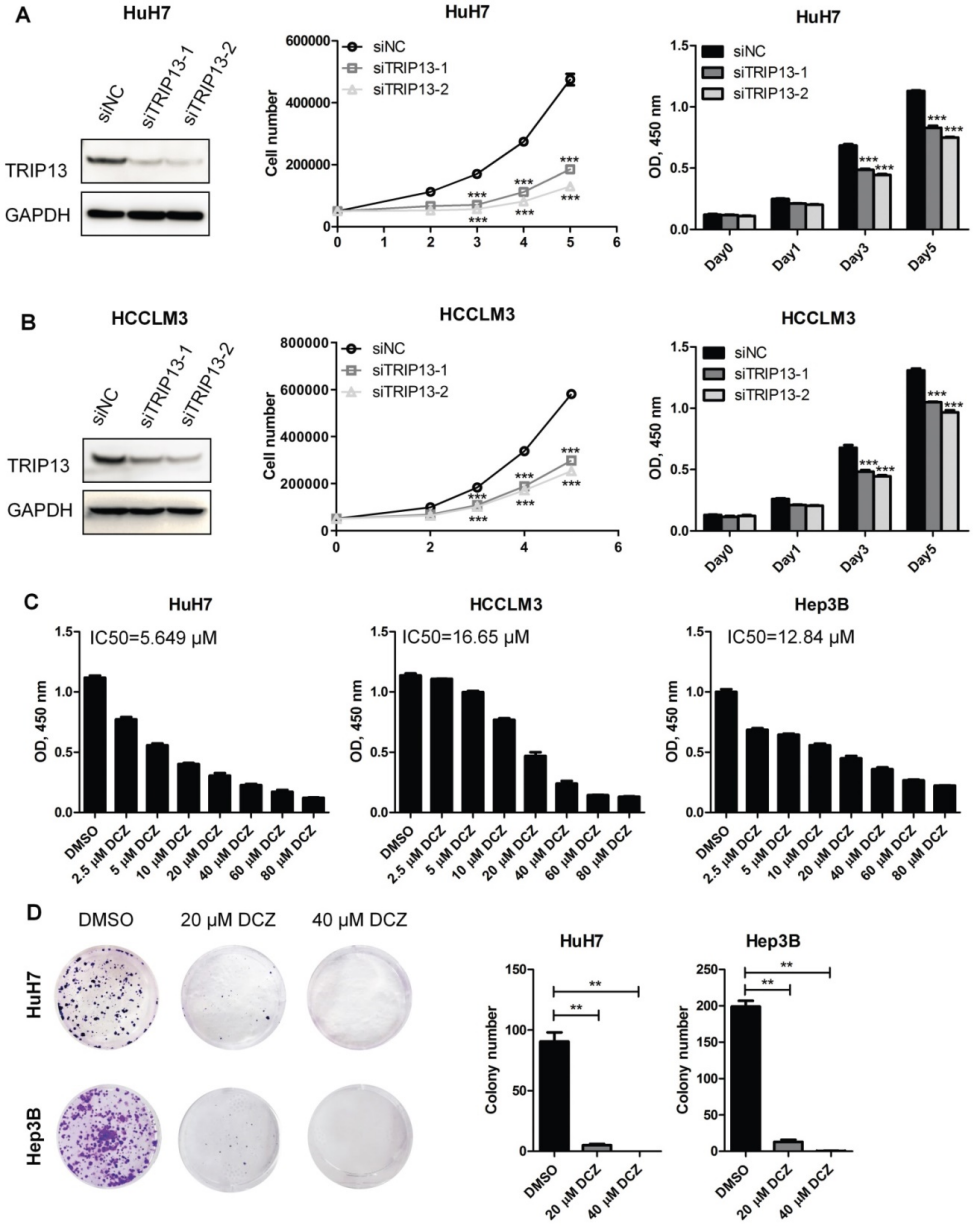
** TRIP13 inhibitor DCZ0415 suppressed the growth of HCC cells.** (A-B) Knockdown of TRIP13 in HCC cells by siRNA, the expression of TRIP13 were assessed by western blot and the growth/viability of cells were measured by trypan blue staining or CCK8 assay; (**C**) HCC cells were treated with different concentrations of DCZ0415, the viability of cells was measured by CCK8 assay; (D) HuH7 and Hep3B cells were treated with DCZ0415 (20 or 40 μM), the growth of cells was detected colony formation assay. Data are shown as mean ±SEM. **p< 0.01, ***p<0.001.

To investigate the therapeutic role of TRIP13 inhibitor DCZ0415 in HCC, we first verify the function of TRIP13 in HCC cell proliferation. As shown in Figure 1 A-B, the Knockdown of TRIP13 in HCC cells (by siRNA) significantly decreased cell proliferation and viability. To confirm that, TRIP13 knockdown HCCLM3 cells (by shRNA) were injected subcutaneously into nude mice. The animal study protocol was approved by and performed following the Institutional Animal Care and Use Committee of Nanjing Medical University (IACUC NMU). We found that the knockdown of TRIP13 dramatically slowed down the growth of HCC tumors (Figure S1A). In addition to these, overexpression of TRIP13 enhanced the growth of HCC tumors in an orthotopic HCC model (Figure S1B). These results demonstrate that TRIP13 promotes the proliferation and tumorigenesis of HCC cells. As an inhibitor of TRIP13, DCZ0415 was then tested whether it is able to inhibit HCC proliferation. As shown in Figure 1C, treatment HCC cells with DCZ0415 significantly reduced cell viability in a dose-dependent manner (IC50 = 5.649 μM for HuH7, IC50 = 16.65 μM for HCCLM3 and IC50 = 12.84 μM for Hep3B). Moreover, colony formation assay also showed that DCZ0415 significantly inhibited HCC cell growth (Figure 1D). Taken together, these data suggest that TRIP13 inhibitor DCZ0415 could suppress the proliferation of HCC cells.

### DCZ0415 induced cell apoptosis and cell cycle arrest in HCC

**Figure 2:**
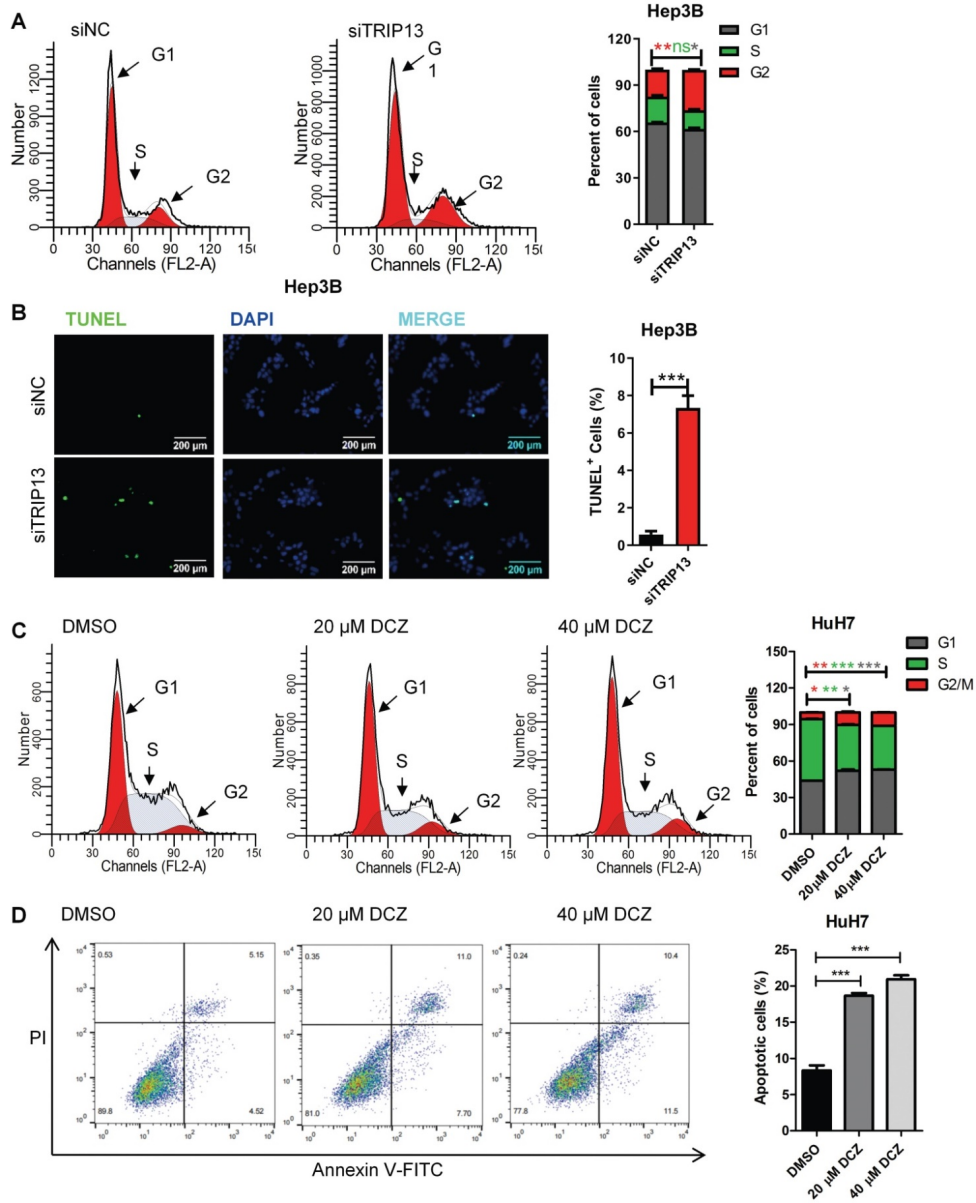
** DCZ0415 induced HCC cells apoptosis and cell cycle arrest.** (A) Flow cytometer analysis of Hep3B cell cycle phases after transfected with TRIP13 siRNA. (B) TUNEL assay analysis of Hep3B cell apoptosis after silencing TRIP13. (C-D) HuH7 cells were treated with DCZ0415 (20 or 40 μM) for 24 h, cell cycle and the percentage of apoptotic cells were analyzed by Flow cytometer. Data are shown as mean ±SEM. *p<0.05; **p<0.01; ***p<0.001; ns, not significant.

Apoptosis resistance and rapid cell division are the main reasons for cancer cell proliferation. To determine the effect of DCZ0415 and TRIP13 on apoptosis and cell cycle in HCC, TRIP13 was knocked down with siRNA or inhibited with DCZ0415. As shown in Figure 2A-B, knockdown of TRIP13 markedly increased the percentage of cells in the G2/M phase and induced cell apoptosis in Hep3B cells. Similarly, inhibition of TRIP13 with DCZ0415 also significantly induced cell cycle arrested at the G2/M phase and increased the percentage of apoptosis in HuH7 cells (Figure 2C-D). These data showed that TRIP13, which was inhibited by DCZ0415, participated in cell death and cell cycle.

### DCZ0415 inhibited the migration and invasion of HCC cells

**Figure 4:**
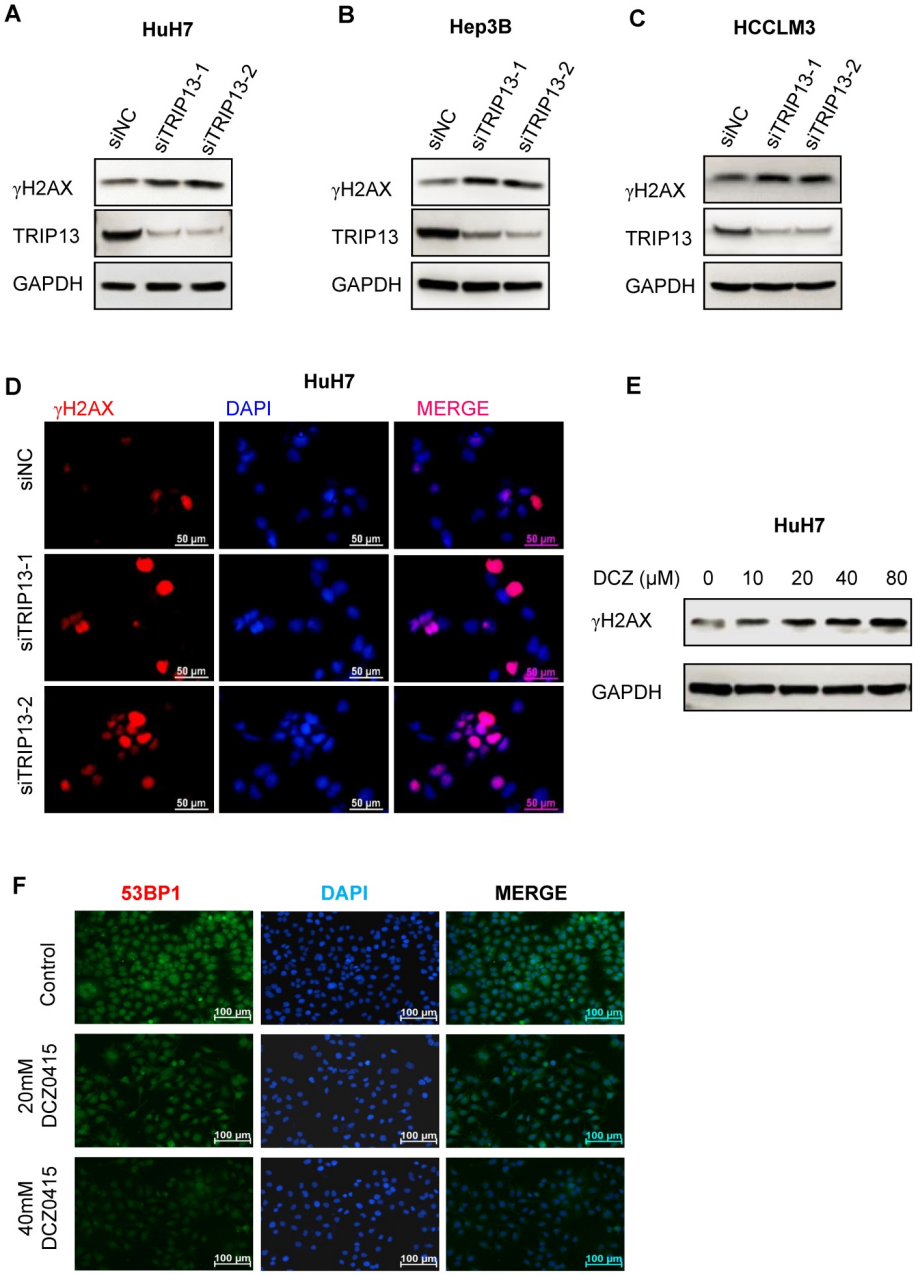
** DCZ0415 suppressed non-homologous end-joining repair.** (A-C) Western Blot analysis of the level of γH2AX after knockdown TRIP13 in HCC cells. (D) Immunofluorescence analysis of γH2AX expression in HuH7 cells after silencing TRIP13. (E) Western blot analysis of γH2AX expression in HuH7 cells after administration with DCZ0415. (F) Immunofluorescence analysis of 53BP1 expression in HuH7 cells after treated by different concentrations of DCZ0415.

Tumor metastasis is one of the major obstacles to improve the prognosis of cancers. It is also the main reason for HCC-associated death 26-29. To evaluate the role of DCZ0415 in the process of HCC cell metastasis, HuH7 cells were treated with DCZ0415 and the motility of the cells was tested by transwell assay. As shown in Figure 4A, DCZ0415 significantly inhibited the migration and invasion of HuH7 cells. Consistent with these, we found that knockdown of TRIP13 in HuH7 cells dramatically suppressed the migration and invasion of the cells. While overexpression of TRIP13 significantly accelerated these processes (Figure 4B-C). Collectively, these results demonstrate that targeting TRIP13 with inhibitor may provide a promising treatment strategy for inhibiting HCC metastasis.

### DCZ0415 impaired non-homologous end-joining repair

TRIP13 was reported to promote non-homologous end-joining repair (NHEJ repair) and induce chemoresistance in head and neck cancer 15. Its inhibitor DCZ0415 also impaired the process of non-homologous end-joining repair 19. To determine whether TRIP13 and DCZ0415 were involved in the process of NHEJ repair in HCC, HCC cells were transfected with TRIP13 siRNA or treated with DCZ0415. As shown in Figure 4A-C, we observed significant increase in levels of γH2AX in HCC cells after silencing TRIP13. Similar results were found by immunofluorescence assay (Figure 4D). In line with these, higher levels of γH2AX were found in DCZ0415-treated HCC cells (Figure 4E). Furthermore, immunofluorescence analysis was performed to understand on which pathway DCZ0415 plays its inhibition's role. In increasing the dosage of DCZ0415, a significant reduction of the fluorescence intensity corresponding to the 53BP1, an NHEJ pathway-specific protein 30, is observed (Figure 4F). These results suggest that loss of TRIP13 or inhibition of TRIP13 impaired the process of NHEJ repair.

### The combination of DCZ0415 and Olaparib synergistically suppressed HCC cell proliferation

**Figure 5:**
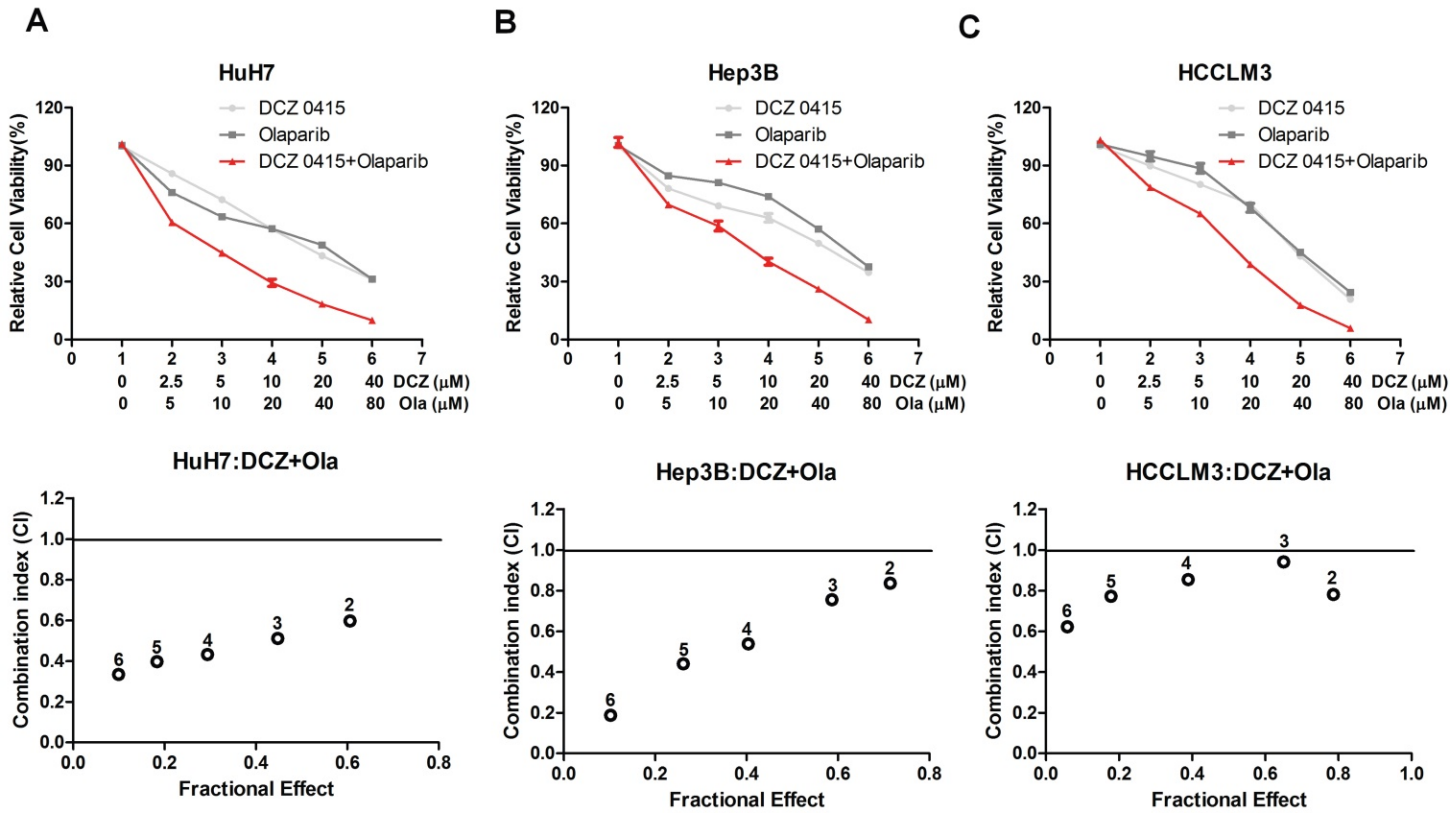
** The effect of DCZ0415 and Olaparib on HCC cell proliferation.** (A-C) HCC cells were treated with or without indicated concentrations of DCZ0415 and Olaparib, the viability of cells was detected by CCK8 assay and the combination index (CI) was calculated by CompuSyn software. CI < 1 was considered synergistic activity.

Previous studies have reported that PARP1 inhibitor Olaparib inhibits many tumor cell growth, like pancreatic cancer 31, prostate cancer 32 and breast cancer 33. In addition, inhibition of PARP1 with Olaparib and DNA-PKcs with NU7441 synergistically suppressed HCC cell survival 24. Considering TRIP13 induced treatment resistance by interacting with NHEJ proteins KU70/KU80/DNA-PKcs and its inhibitor DCZ0415 impaired NHEJ 15, 19, we speculated that the combination of DCZ0415 and Olaparib increased the inhibition level of HCC. As shown in Figure 5A-C, compared with single-drug treatment group, including Olaparib and DCZ0415, combination group exhibited higher inhibition level in HCC cells, which indicated that DCZ0415 increased Olaparib efficiency in HCC cells, combination with TRIP13 inhibitor DCZ0415 and PARP1 inhibitor Olaparib may be a potential therapy for HCC.

### TRIP13 is regulated by transcription factor SP1

**Figure 6:**
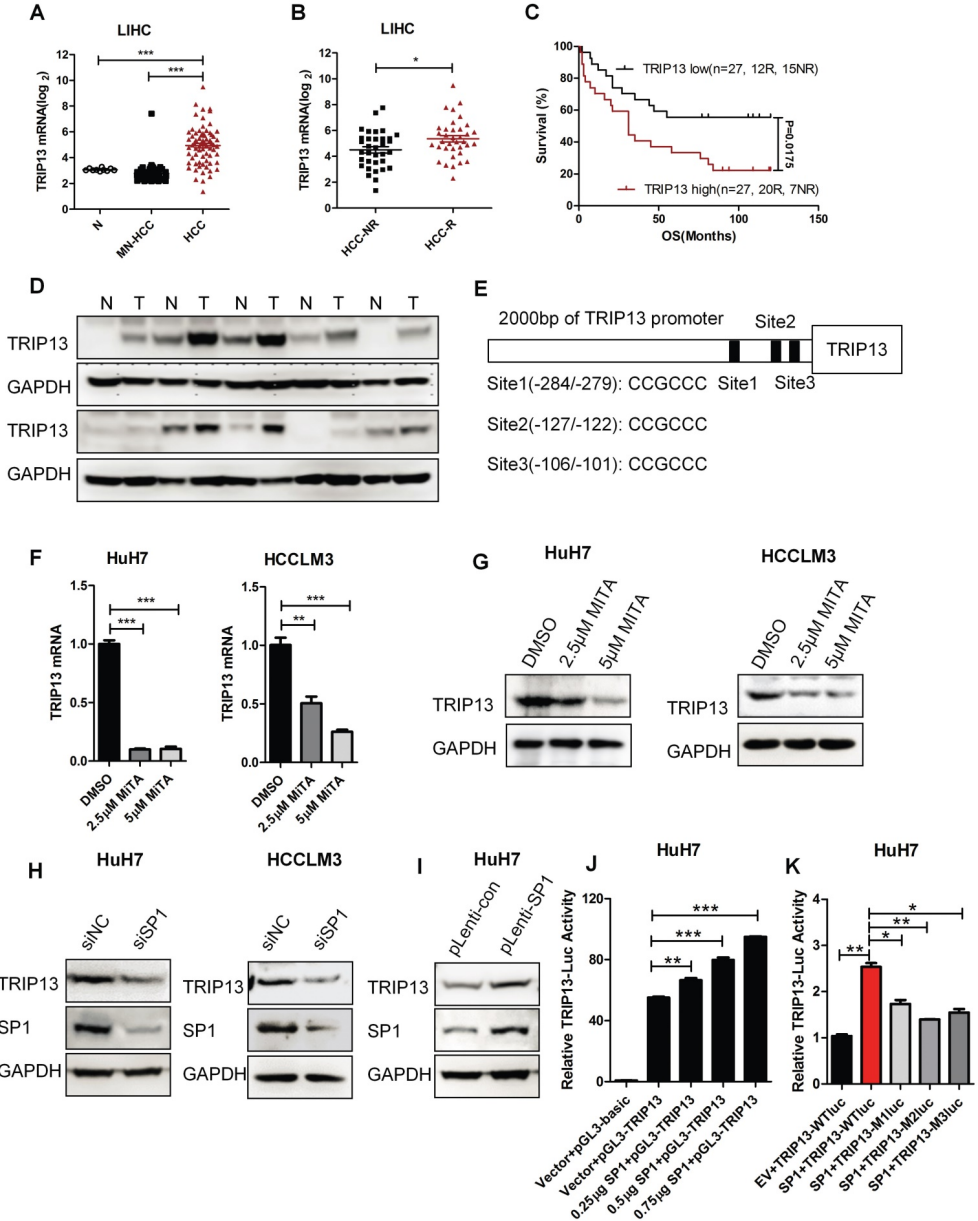
** The expression of TRIP13 in HCC is regulated by SP1.** (A) The expression of TRIP13 in normal liver tissues (N), matched normal livers of HCC patients (MN-HCC) and HCC groups were analyzed according to global gene expression profile database; (B) TRIP13 expression in no-recurrence (HCC-NR) and recurrence HCC (HCC-R) groups were analyzed by microarray; (C) Expressions of TRIP13 was associated with overall survival and recurrence in HCC patients; (D) Western blot assay assessed the expression of TRIP13 in human HCC tissues (T) match normal tissues (N); (E) The predict binding sites of SP1 in the upstream region of TRIP13. RT-qPCR (F) or Western blot (G) analysis of TRIP13 expression level after management with SP1 inhibitor, MITA (2.5 or 5 μM) for 24 h; (H-I) Western blot assay detected expression of TRIP13 after knockdown or overexpression of SP1; (J) pGL3-TRIP13-promoter, SP1 and renilla plasmids were co-transfected into HuH7 cells, luciferase activities were measured with a dual-luciferase reporter assay system; (K) pGL3-TRIP13-promoter or mutant pGL3-TRIP13-promoter (M1, M2, or M3) were co-transfected with SP1 and renilla plasmids into HuH7 cells, luciferase activities were measured with a dual-luciferase reporter assay system. Data are shown as mean ±SEM. *p<0.05, ** p<0.01, ***p<0.001.

TRIP13 was reported overexpressed in HCC 18. However, its role in early recurrence and the underlying mechanisms of overexpression in HCC are still unknown. Our analysis results revealed that the expression of TRIP13 was upregulated in HCC and had a positive correlation with early recurrence (Figure 6A-D) 34, 35.To further investigate the upstream molecular mechanism that caused the up-regulation of TRIP13 in HCC, we analyzed the promoter of TRIP13 through JASPAR and ALGGEN-PROMO databases. We identified that transcription factor SP1 has three binding sites upstream of the TRIP13 transcription initiation site (Figure 6E). Furthermore, we used the inhibitor of SP1, Mithramycin A (MITA), to test its relationship with TRIP13. As shown in Figure 6F-G, MITA reduced TRIP13 mRNA and protein levels in HCC cells. Moreover, using SP1 siRNA, TRIP13 expression also decreased in HCC cells, while overexpression of SP1 increased the protein level of TRIP13 in HuH7 cells (Figure 6H). We further transfected extra SP1 into HuH7 cells and found that TRIP13 promoter-luciferase activities were increasing with the increased concentration of SP1 compared with the control vector (Figure 6J). However, mutated SP1 binding sites in TRIP13 promoters impaired the luciferase activity in HuH7 cells (Figure 6K). Taken together, our results demonstrate that TRIP13 may be a potential diagnostic marker for HCC with early recurrence and the expression of TRIP13 in HCC was regulated by SP1.

## Discussion

Increasing evidence verified that TRIP13, one thyroid hormone receptor-interacting factor, is closely related to many tumors' development and progression 12, 15, 17, 19. Yao and co-authors reported that TRIP13 was a high expression in HCC tissues and greater TRIP13 predicted a worse diagnosis. In addition, knockdown of TRIP13 attenuated tumorigenesis, including HCC. TRIP13 over-expression induced HCC migration and invasion 36, which is consist of our study. DCZ0415 is the special target drug of TRIP13 19, which was reported in 2018. In our study, we found that DCZ0415 inhibited HCC cell proliferation, migration, and invasion. The phenotype results were similar to silencing TRIP13. Furthermore, we investigated cell apoptosis and cell cycle with flow cytometry assay. DCZ0415 and knockdown of TRIP13 promoted HCC cell death and arrested cell cycle at G2/M phase.

Lots of studies showed that some anticancer drugs activated cell death via regulating DNA damage and repair. DCZ0415 has been tested that enhanced DNA damage and delayed DNA repair 19. γH2AX is closely related to DNA double-strand breaks (DSBs) and can be used as a marker for double-strand repair 37. In this study, we investigated that TRIP13 regulated γH2AX activation both in silence assay and DCZ0415 treatment.

Drug resistance is the biggest obstacle to the later treatment of HCC cases 38. Olaparib, one inhibitor of PARP1, is used to treat a variety of cancers linked to defective BRCA gene, has been testified that could prohibit HCC cell growth synergistically with DNA-PKcs inhibitor 24. TRIP13-/- cells are more sensitive to Olaparib than normal cells 24. Thus, to understand the synergistic effects of DCZ0415 and Olaparib, we tested the effects of DCZ0415 combination with Olaparibto treat HCC cells. Compared with a single treatment of Olaparib, combination groups in HCC cell lines enhanced more growth inhibition, which indicated that combination with TRIP13 inhibitor DCZ0415 and PARP1 inhibitor Olaparib might be a potential therapy for HCC.

Furthermore, we investigated SP1 as a positive transcription factor of TRIP13. Recently reports showed that SP1 is a higher expression in HCC and correlation with HCC prognosis. Li et al. investigated that targeting the SP1-TERT axis for tumor growth in HCC 39. SP1 mediated USP33/c-Met expression to facilitate metastasis in HCC 40. Our previous study also showed that SP1 induced the expression of STK39 and then promoted the progression of HCC 41. In this study, to explore the underlying mechanisms of TRIP13 overexpressed in HCC, promoter regions of TRIP13 were analyzed with JASPAR and PROMO databases. We found three SP1 binding regions upstream to the TSS of the TRIP13. Inhibition or knockdown of SP1 significantly inhibited the expression of TRIP13, while overexpression of SP1 dramatically promoted TRIP13 expression. Moreover, dual-luciferase reporter assay revealed that mutation of SP1 binding regions impaired SP1-induced TRIP13 transcript activity. Thus, our data verified that the expression of TRIP13 in HCC is regulated by SP1. Existing studies have been reported that the target for anti-HCC TRIP13 is contained a transcription factor protein OCT4 (Octamer-binding transcription factor 4) binding motifs in its promoter region 42. OCT4 is thus positioned to participate in the HCC tumorigenesis and progression and could be a potential drug target for HCC. Meanwhile, this study found that SP1 could also play a regulatory role in TRIP13 expression. This result suggests that SP1/TRIP13 axis sounds another approach as a potential therapy for anti-HCC.

In conclusion, our present data indicated that inhibitors targeting TRIP13 decreased the proliferation, migration, and invasion of HCC cells, inhibition of TRIP13 induced cell death and cell cycle arrest via suppressing DSB repair. TRIP13 inhibitor DCZ0415 had a synergistic effect with PARP1 inhibitor Olaparib in HCC, which might provide a prospective therapy for the treatment of HCC. The highly expressed TRIP13 in early recurrent HCC tumors revealed that TRIP13 might be a potential diagnostic marker for HCC with early recurrence.

## Supplementary Material

Supplementary figure.

## Figures and Tables

**Figure 3:**
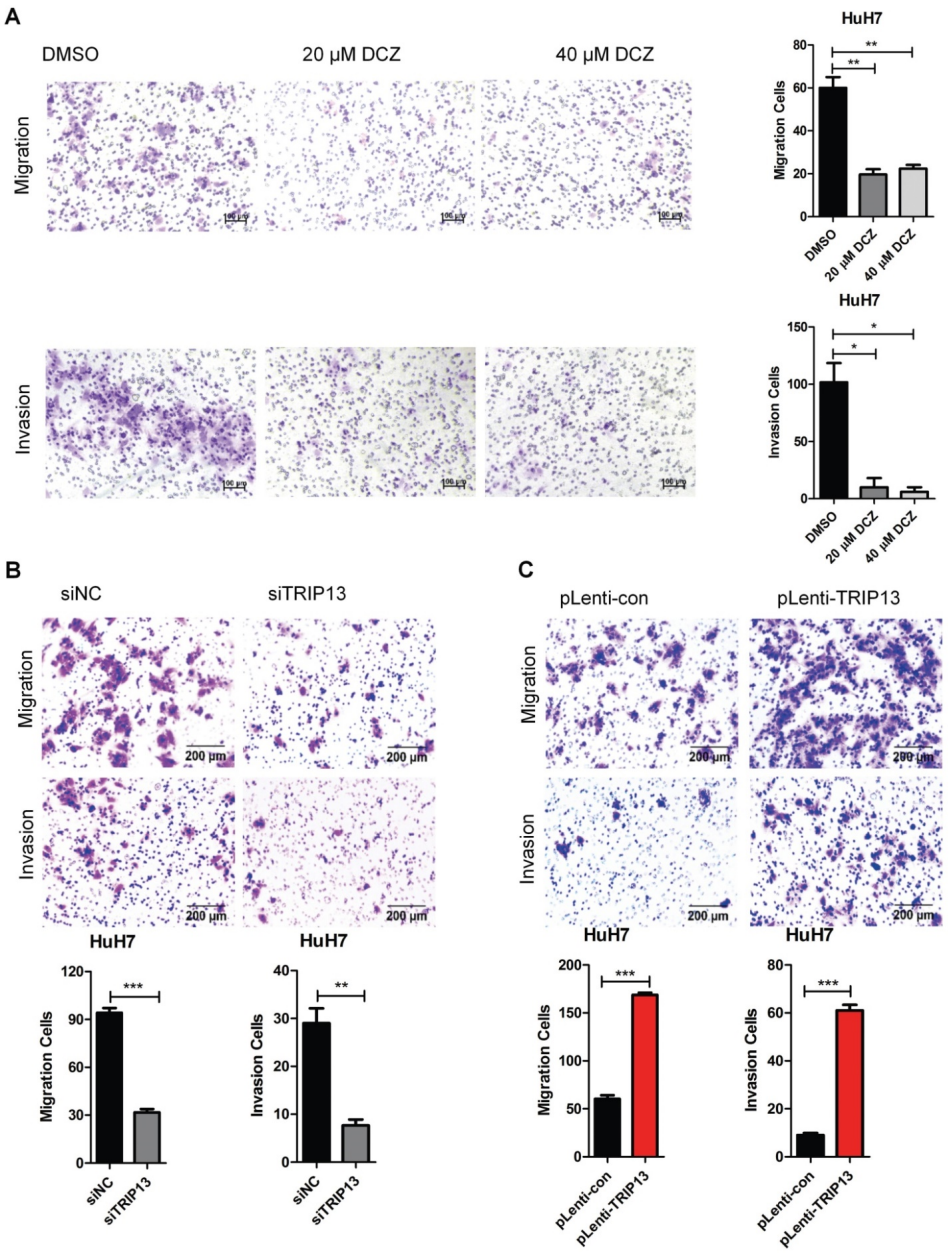
** DCZ0415 inhibited the migration and invasion of HCC cells.** (A) HuH7 cells were treated with DCZ0415 (20 or 40 μM) for 36 h, migrated or invasive cells were measured by transwell assay; (B) Knockdown of TRIP13 in HuH7 cells by siRNA, the migration ability and invasion ability of cells were measured by transwell assay; (C) Overexpressing TRIP13 in HuH7 cells, the migration ability and invasion ability of cells were assessed by transwell assay. Data are shown as mean ±SEM. *p<0.05; **p<0.01; ***p<0.001.

## Abbreviations

HCC: hepatocellular carcinoma
TRIP13: Thyroid hormone receptor interactor 13
SAC: spindle assembly checkpoint
NHEJ: non-homologous end joining
ATCC: American Type Culture Collection
TUNEL: terminal deoxynucleotidyl transferase dUTP nick end labeling
CIN: chromosomal instability
