# Supplementary material for: Inducing Synergistic DNA Damage by TRIP13 and PARP1 Inhibitors Provides a Potential Treatment for Hepatocellular Carcinoma
Source: J Cancer. 2022 Apr 11;13(7):2226–37. doi: 10.7150/jca.66020 (PMC9066198; doi:10.7150/jca.66020)

Inducing Synergistic DNA Damage By TRIP13 And PARP1 Inhibitors Provides A Potential Treatment For Hepatocellular Carcinoma

Supplementary Figure 1. TRIP13 promoted the growth of HCC *in vivo*. (A)TRIP13-knockdown (shTRIP13) or negative control (shNC) HCCLM3 cells were inoculated subcutaneously into NOD-SCID mice. Tumor sizes were measured every week. After 5 weeks of injection, the tumors were photographedand recorded theirweights. (B) HCCLM3 cells, including overexpression TRIP13 (pLenti-TRIP13) and matched normal (pLenti-con), were orthotopic injected in liver tissues after injected 5 weeks, mice have been tested the luciferase signals for each group. Data are shown as mean ±SEM. *$p<0.05$, ***$p<0.001$.

Figure S1

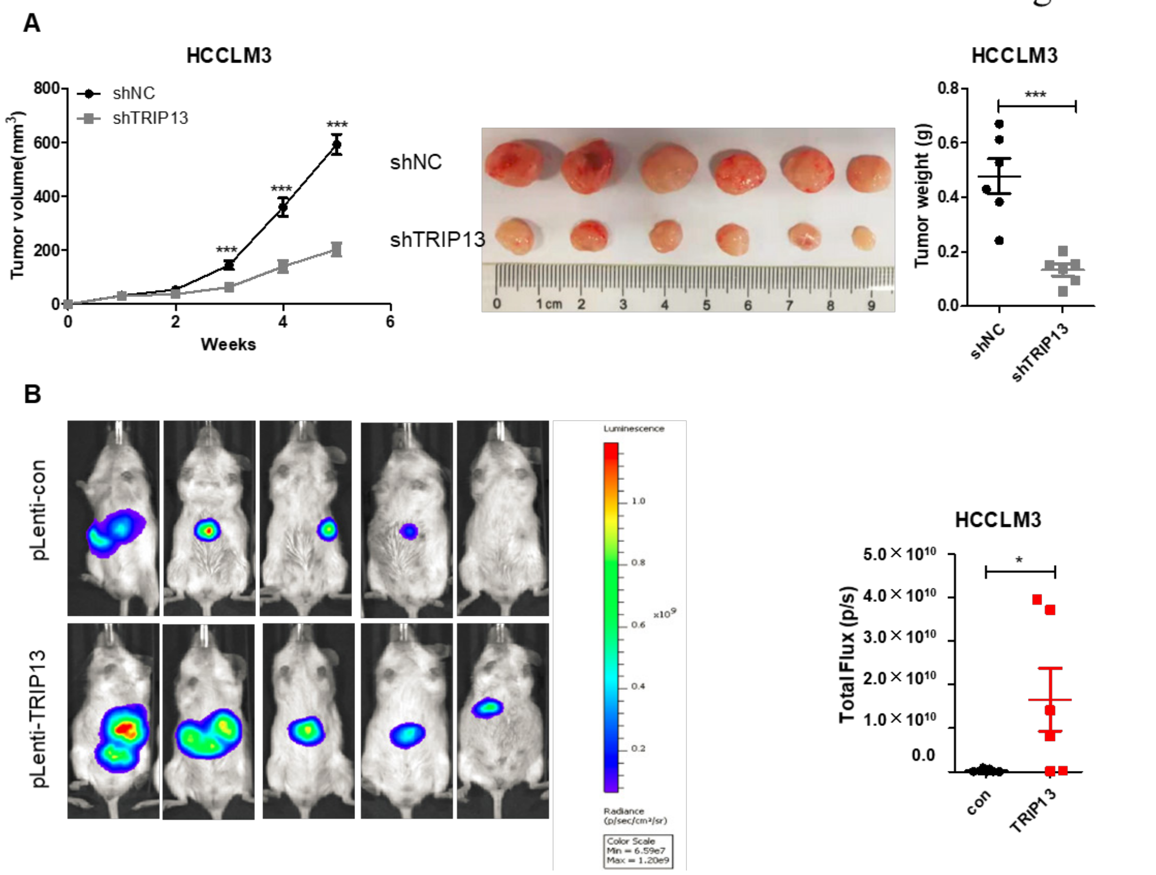

Supplement: Supplementary file 1 — Supplementary figure. [file jcav13p2226s1.pdf]
